# Arrhythmogenic cardiomyopathy: double, double toil, and trouble

**DOI:** 10.1093/ehjcr/ytaf563

**Published:** 2025-11-05

**Authors:** Joana Certo Pereira, Oana Moldovan, Pedro Lopes, Bruno ML Rocha

**Affiliations:** Cardiology Department, Hospital de Santa Cruz (ULSLO), Avenida Professor Doutor Reinaldo dos Santos 2790-134, Carnaxide, Lisbon, Portugal; Genetics, Hospital de Santa Cruz (ULSLO), Avenida Professor Doutor Reinaldo dos Santos 2790-134, Carnaxide, Lisbon, Portugal; Cardiology Department, Hospital de Santa Cruz (ULSLO), Avenida Professor Doutor Reinaldo dos Santos 2790-134, Carnaxide, Lisbon, Portugal; Cardiac Imaging, Hospital de Santa Cruz (ULSLO), Avenida Professor Doutor Reinaldo dos Santos 2790-134, Carnaxide, Lisbon, Portugal; Cardiology Department, Hospital de Santa Cruz (ULSLO), Avenida Professor Doutor Reinaldo dos Santos 2790-134, Carnaxide, Lisbon, Portugal; Cardiomyopathy Unit, Advanced Heart Failure and Heart Transplant, Hospital de Santa Cruz (ULSLO), Avenida Professor Doutor Reinaldo dos Santos 2790-134, Carnaxide, Lisbon, Portugal

**Keywords:** Arrhythmogenic cardiomyopathy, Cardiac magnetic resonance, Triangle of dysplasia, Genetic testing, Sudden cardiac death

## Summary

While single genetic variants associated with arrhythmogenic cardiomyopathy (ACM) have a highly variable penetrance and expression, compound heterozygosity increases the likelihood of displaying the ACM phenotype.^1^ We here describe the case of a patient with a positive screening with double mutation and a late-presenting ACM phenotype, emphasizing the importance of a structured systematic cascade screening.

## Case description

**Figure 1:**
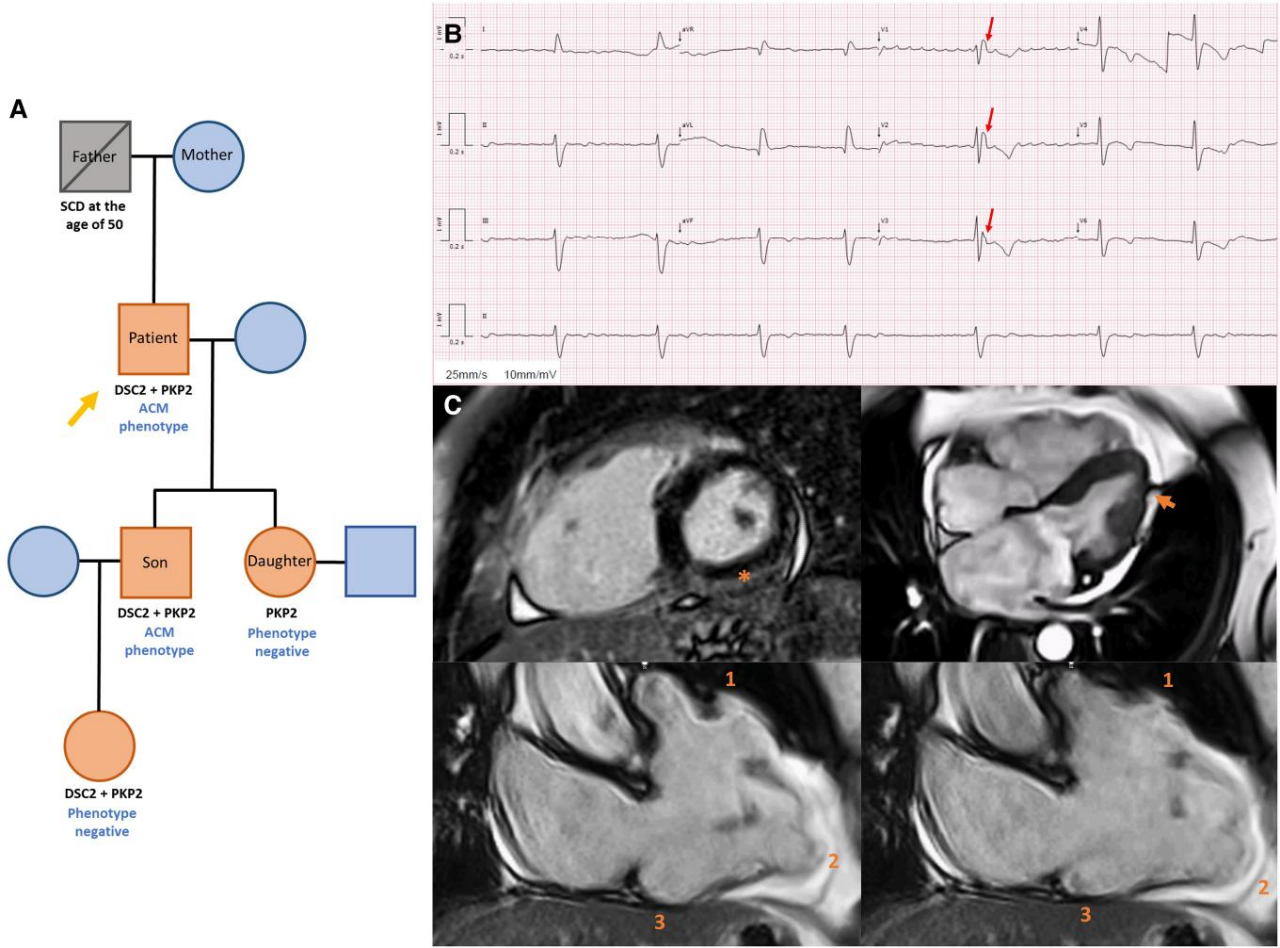
(*A*) Family tree depicting the members with pathogenic mutations, with and without an ACM phenotype. The patient’s son exhibits a positive ACM phenotype, including RV dilation and systolic dysfunction with biventricular extensive LGE, and has an implantable cardioverter-defibrillator for secondary prevention, following a syncope due to monomorphic ventricular tachycardia with left bundle branch block morphology. (*B*) ECG of the 70-year-old patient depicting atrial fibrillation (50–60 b.p.m.), complete right bundle branch block, epsilon waves in V_1_–V_3_ (arrow), and T-wave inversion in all precordial leads. (*C*) Cardiac magnetic resonance showing subepicardial LGE in the inferolateral and inferior walls of the left ventricle (asterisk), irregular myocardial contour of the LV lateral wall [‘rat-bite sign’ (arrow)], and dyskinetic movement of the right ventricular apex, and the inflow and outflow tracts [ACM ‘triangle of dysplasia’ (1, 2, 3)].

A 70-year-old man patient with permanent atrial fibrillation, hypertension, and type 2 diabetes was referred to the Cardiomyopathy Clinic following genetic cascade screening for ACM. His son (index case) had symptomatic ACM with two pathogenic variants: PKP2 (c.1170 + 2T > A) and DSC2 [c.2112_2116del p.(Phe708fs*14)]. The patient also had both the same mutations. His father had died suddenly at the age of 50 years, of unknown cause, and two other relatives were genotype-positive but phenotype-negative—see the genogram (*Figure 1A*).

He complained of mild fatigue and orthostatic intolerance. The ECG (*Figure 1B*) showed atrial fibrillation, complete right bundle branch block, and epsilon waves in V_1_–V_3_. The transthoracic echocardiography revealed right ventricular (RV) dilation and moderate systolic dysfunction with free wall akinesia. The cardiac magnetic resonance imaging (*Figure 1C*; Supplementary Material Supplementary material online, *Videos S1*-*S4*) confirmed the reduced RV systolic function (ejection fraction 30%), apical, inflow, and outflow dyskinesia and subepicardial late gadolinium enhancement (LGE) in the so-called triangle of dysplasia. Late gadolinium enhancement was also seen in the left ventricular infero-lateral wall. Notably, both ventricles had myocardial contour irregularities (‘rat-bite sign’), suggestive of adipose infiltration. The 48 h Holter monitor (while on bisoprolol 5 mg) recorded 73 polymorphic premature ventricular contractions.

The diagnosis of biventricular ACM was established (according to the 2020 Padua criteria^2^). The estimated 5 year arrhythmic risk was 17.3% using the ACM risk calculator v3.0.^3^ Accordingly, we recommended an implantable cardioverter-defibrillator, which was placed for the primary prevention of sudden cardiac death. At 1.5 years of follow-up, the patient complains of mild fatigue and has had no cardiovascular events or ICD therapies.

## Supplementary Material

ytaf563_Supplementary_Data

## Data Availability

The data underlying this article are available in the article and in its online Supplementary material.
