# Supplementary material for: Arrhythmogenic cardiomyopathy: double, double toil, and trouble
Source: Eur Heart J Case Rep. 2025 Nov 5;9(11):ytaf563. doi: 10.1093/ehjcr/ytaf563 (PMC12626129; doi:10.1093/ehjcr/ytaf563)
Supplement: ytaf563_Supplementary_Data [file ytaf563_supplementary_data.zip › Supplementary material.docx]

**Supplementary Material - Videos**

**Video 1** – Cardiac magnetic resonance, cine acquisition of the three-chamber view, showing irregularities of the epicardial contour (“rat-bite sign”) of the posterior wall of the left ventricle.

**Video 2** – Cardiac magnetic resonance, cine acquisition of the four-chamber view, showing irregularities of the epicardial contour (“rat-bite sign”) of the anterolateral wall of the left ventricle.

**Video 3** – Cardiac magnetic resonance, cine view of the right ventricular outflow tract, showing segmental dyskinesia of the RV outflow tract.

**Video 4** – Cardiac magnetic resonance cine sequence demonstrating dyskinesia of the right ventricular apex, and the inflow, and outflow tracts, corresponding to the ACM “triangle of dysplasia”.
